# Collaborative Irrationality, Akrasia, and Groupthink: Social Disruptions of Emotion Regulation

**DOI:** 10.3389/fpsyg.2016.02002

**Published:** 2017-01-04

**Authors:** Thomas Szanto

**Affiliations:** Department for Media, Communication and Cognition, Center for Subjectivity Research, University of Copenhagen Copenhagen, Denmark

**Keywords:** collaborative irrationality, emotion regulation and dysregulation, emotional co-regulation, interpersonal emotion regulation, akrasia, self-deception, groupthink, group identification

## Abstract

The present paper proposes an integrative account of social forms of practical irrationality and corresponding disruptions of individual and group-level emotion regulation (ER). I will especially focus on disruptions in ER by means of collaborative agential and doxastic akrasia. I begin by distinguishing mutual, communal and collaborative forms of akrasia. Such a taxonomy seems all the more needed as, rather surprisingly, in the face of huge philosophical interest in analysing the possibility, structure, and mechanisms of *individual* practical irrationality, with very little exception, there are no comparable accounts of social and collaborative cases. However, I believe that, if it is true that individual akrasia is, in the long run, harmful for those who entertain it, this is even more so in social contexts. I will illustrate this point by drawing on various small group settings, and explore a number of socio-psychological mechanisms underlying collaborative irrationality, in particular groupthink. Specifically, I suggest that in collaborative cases there is what I call a *spiraling of practical irrationality* at play. I will argue that this is typically correlated and indeed partly due to biases in individual members’ affect control and eventually the group’s with whom the members identify.

## Introduction

People not only have emotions, they also regulate them. In regulating emotions, we select and adjust the situations of affective import, or modulate our attention or behavioral responses (Gross, 1998). It is widely agreed that the way an emotion is experienced closely reflects the way in which it is regulated (Frijda, 1986; Krueger, 2016). But emotion regulation (ER) does not occur in a social void. Rather, it is deeply embedded in and modulated by social interaction, social identity, or group membership. Indeed, the cognitive aspects of interactional and sociocultural influences in emotional co-regulation have received considerable attention (e.g., Eisenberg et al., 1998; Eisenberg and Spinrad, 2004; Mesquita and Albert, 2007; Hofer and Eisenberg, 2008; von Scheve, 2012; De Leersnyder et al., 2013). However, the broad range of potential disruptions and, in particular, the collaborative forms these disruptions often assume, has been very much sidelined and little understood.

The present paper aims to fill this gap by focusing on social forms of practical irrationality. Specifically, I will concentrate on two potential disruptions in ER, namely social forms of agential akrasia (AA) and doxastic akrasia (DA). On a first approximation, AA consists of acting against one’s own better judgment or against some relevant set of values, norms or reasons, or in performing an action that runs counter to one’s intention. Doxastic akrasia, a variant of self-deception, occurs if one believes something against one’s own better reasons or epistemic standards, or ‘in the teeth of evidence.’

Against this background, the paper pursues two objectives: (1) First, I will argue that specific collaborative forms of AA and DA are possible, and propose a novel model to analyze them. This seems to be a crucial task. After all, in the face of great philosophical interest in analysing the possibility, structure and mechanisms of *individual* practical irrationality, with little exception concerning self-deception (Harré, 1988; Ruddick, 1988; Tenbrunsel and Messick, 2004; Deweese-Boyd, 2010) and even less akrasia (Pettit, 2003b), there are surprisingly no comparable accounts of social and *collaborative* irrationality. However, I not only contend that these are common phenomena; moreover, I believe that, if it is true that individual practical irrationality is in the long run harmful for those who entertain it, this is even more so in collaborative cases (Goleman, 1989). (2) Secondly, I shall argue that collaborative engagements often play a contributing or even constitutive role in entering or maintaining practical irrationality. I argue that this is often correlated to ER-biases and to a large extent indeed due to the disruptive role of collaborative irrationality on ER. Specifically, I will suggest that it is largely due to socially biased, motivated misidentification of one’s own affects, which eventually biases one’s affect control and also the ER-mechanisms of one’s group. Finally, I shall show how in a feedback-loop that I call collaborative spiraling of irrationality this ultimately reinforces the irrational tendencies of the respective parties.

The paper is organized as follows: I begin by fleshing out the concepts of AA and DA and propose three requirements that agents capable of such irrationalities must fulfill (see Section “Self-deception and Doxastic Akrasia”): the intentionality, the minimal rationality, and the overall rational integrity requirement. Next, I outline the key mechanisms and (social) disruptions of ER. In particular, I suggest that DA inhibits central features of successful ER: subjects’ clarity about the type and the evaluative or cognitive content of a given emotion, or even their basic awareness of having a certain type of emotion (see “Emotion Regulation and Its Social Biases”). I then explore practical irrationality in various social contexts and their correlation with ER-disruptions. I distinguish mutual, communal and collaborative forms of social irrationality (SI), and explore especially the collaborative cases. I will mainly draw on the case of a clinical smoking therapy group and demonstrate how ‘groupthink’ (Janis, 1982) modulates even such allegedly purely physiological arousal patterns like those induced by nicotine.^1^ In this section, I resume the issue of dysregulation and focus on the corruption of group-level ER. Here, I will also discuss some further socio-psychological mechanisms, which account for the emergence of SI in deliberative groups, notably group-polarization, choice shift and the pooling of unshared information (see “The Collaborative Spiraling of Irrationality”). Finally, I provide a conceptual explanation of collaborative irrationality in terms of group identification. Drawing on the overall rational integrity requirement, I claim that what happens is a failure of integrating first-person *singular* and first-person *plural* rational point of views, while maintaining group identification (see “Explaining Collaborative Irrationality”). I conclude by pointing to some directions for future research (see “Conclusion and Future Directions”).

## Self-Deception and Doxastic Akrasia

There has been much debate as to whether synchronous forms of practical irrationality are possible at all. The question is whether one can synchronously hold contradictory beliefs as to what would be best to do. The issue poses itself with particular force when it comes to the role of emotions. The issue is neither that an agent, under the influence of the emotion at the time of the practical deliberation, changes her view about what it would be best to do; nor do we necessarily have to accept the view that emotions can be so powerful as to directly change the behavior of an agent at the very instance of the respective action, or to refer to ‘irresistible desires’—a notion that some have rightly rejected (Watson, 1977; Elster, 2010). Rather, the issue is that emotions influence or motivate an action that is, at the time of its execution, contrary to the agent’s beliefs about what, all things considered, is best to do.

Thus, some have suggested that emotions will only “cloud or bias” the cognitive processes (information-gathering, etc.) upon which the agent’s practical deliberation is based, or influence the agent’s rational choice. The—subsequent—irrational action is then due to a (temporary) ‘preference reversal’ (Elster, 2006). As we will see, I agree with those accounts that argue that reference to a ‘partition of the mind’ in the irrational agent as prominently suggested by Davidson (1982), is in such cases “little more than hand waving” (Elster, 2010, p. 270). However, I believe that by the same token mere reference to preference reversal over time or diachronic accounts of practical irrationality are dangerously close to reducing practical irrationality to a ‘change of mind’ and will not do either.^2^ Thus, we need an account that captures the tension arising from holding synchronic contradictory beliefs, desires or reasons for action. Such an account is provided by what Mele (1987) has aptly labeled “last-ditch” cases of practical irrationality. In the following, I will rely on last-ditch cases and assume that they are psychologically possible and real.

Here, then, is what last-ditch AA and DA amount to: Assume a subject S who is a non-pathological rational believer under normal circumstances (S doesn’t have severe Alzheimer, is not a drug-addict, is not hallucinating, unconscious, etc.) who attends more or less strictly, but given normal epistemic standards, to logical and rational consistency. For now, such an admittedly liberal, rough-and-ready characterization of rationality will suffice; I will come back to that below, however.

(AA) An action A is strictly akratic iff

(1) S is an intentional agent such that S intentionally will A at *t* only if S judges that A-ing at *t* is all-things-considered better than B-ing at *t*, and S believes that she can either A or B at *t* (or be given the alternate possibility of not A-ing at *t*);
(2a) S holds a belief at *t* to the effect that all things considered she has sufficient reason for her not A-ing at *t* (or for doing B, where B is incompatible with doing A);
(2b) Based on the evaluation of A in attaining S’s goal at *t*, S decisively judges that it is best not to A at *t*;
(3) S (intentionally) A-s at *t*.

A number of technicalities set aside, and however, differently one might then wish to explain how rational agents are led from (1) to (3), or how AA is possible at all, this fairly mirrors the standard picture of what synchronic AA would amount to (Davidson, 1970; Bratman, 1979; Pears, 1984; Mele, 1987; Walker, 1989).

Consider now the similar case of DA, sometimes also called “incontinent belief” (Mele, 2001). Here, we have a motivated case of believing something against one’s own better reasons, assuming, again, a reasonably rational, non-pathological subject.

(DA) S is subject to doxastic akrasia, or holds or retains an akratic belief, iff

(1) S believes that *p* and *q* are incompatible;
(2) S has a reason R to believe that *p*;
(3) S acknowledges that R is a stronger reason than an alternative set of R^∗^s, which warrant *q* (where R and R^∗^s are warranted by all the evidence available to S relevant to *p* and *q*, respectively);
(4) S believes that *q*.

Note that (DA) shares almost all features with another phenomenon of practical irrationality, namely self-deception, except for the fact that both *p* and *q* may be true propositions, whereas according to the standard view, in self-deception, *q* will be false, and S knows or at least takes *q* to be false (Heil, 1984; Mele, 1987). But, as this is the only relevant difference between DA and self-deception proper, most of what I shall discuss below will hold for both DA and self-deception.

Given these definitions, let me now come back to the issue of an alleged partition of mind, which according to some explains what happens in AA and DA. It is crucial to get this point right to appreciate the very force of the tension that subjects are confronted with when engaging in irrational beliefs and action. And more importantly for our present purposes, it is a good starting point to discuss cases of AA and DA where we have interpersonal and collaborative forms of irrationality. Note that is seems to make no sense to speak of inconsistency, let alone irrationality, if what we have is just a conflict between sets of reasons or beliefs *partitioned* or *distributed across* two or more agents or believers. In marked contrast, in usual cases, the subject having a true or sufficiently warranted belief and the subject of self-deception, akrasia or DA are essentially identical: it is an individual subject. However, many have argued that the best or only possible explanation of how agents can arrive at the conclusion of AA or DA—without leading to irresolvable paradoxes—is to assume a multiplicity of rational centers within individuals. The conflicts or inconsistencies are then construed with reference to different aspects in terms of hierarchies of values, epistemic imbalances, non-alignment between motivational strength and evaluative judgments, or a gap between conative and rational poles or cognitive subdivisions (Wiggins, 1978/1979; Davidson, 1982, 1986; Pears, 1984, 1985; Rorty, 1985; Mele, 1987). The details need not concern us here; what is important is that practical irrationality is construed here as a fragmentation or partitioning of “rational homunculi” (De Sousa, 1976) *within individuals*.

Whether or not one subscribes to such homunculi views or rather argues for anti-partitioning accounts (Bach, 1981; Ruddick, 1988; Talbott, 1995; Barnes, 1997; Johnston, 1988), the issue does not hinge on whether one conceives of practical irrationality as a *conflict*, for example, between emotional and doxastic contents or as involving outright *contradiction* between incompatible judgments (Döring, 2008, 2009). Though I clearly favor the first view, in both cases, accounts that refer to a partitioning of rational and/or affective faculties of agents cannot do justice to the sense in which practical irrationality involves a certain tension or precisely a conflict *within one and the same* agent.

To be sure, the concept of the identity of agents must be construed differently in cases in which we have, on the one hand, ordinary individual agents considered by themselves, and those where we have an interpersonal or a collaborative context with a plurality of agents, on the other. Thus, even if one does not accept anti-homuncular and anti-partitioning arguments as conclusive, the question is still how we can capture the conflict in practical irrationality when we *start* with a *plurality* or *collective* of individuals. After all, in such cases, we would normally simply speak of some conflict of interest or social conflict, which is an all-too ordinary phenomenon. In order to address this question, I shall propose three requirements that agents capable of practical irrationality must fulfill, to wit, requirements that both individual as well as a collective of agents, deliberating and acting upon an integrated or unified point of view, can fulfill.

(1) The first is a standard requirement of agency, accepted by almost all philosophers of mind and action today, concerning practical and theoretical intentionality. It amounts to claiming that an (individual or collective) agent capable of any form of practical irrationality (akrasia, DA, or self-deception) must be an intentional agent. The general idea is fairly straightforward. A subject S is an intentional agent, i.e., S is the bearer of intentional properties. S can over time and under various practical and epistemic circumstances form, hold, and robustly entertain intentional attitudes and beliefs with propositional or some otherwise specified intentional content, practical intentions, and/or desires or other motivational, so-called ‘pro-attitudes.’ Moreover, it is these intentional properties that figure in folk-psychological explanations of S’s behavior. Call this the *Intentionality Requirement*.

(2) The second requirement builds directly on this ability of agents entertaining intentional states, but infuses them with certain inferential norms or a minimal form of rationality. Call this the *Minimal Rationality Requirement*. According to this, S will not only have intentional states, but will typically hold relatively consistent, or at least non-contradictory, beliefs, aesthetic, moral, etc., attitudes, rank her preferences, and attend to them and their transitivity (e.g., if S prefers A to B and C to B, then S will also prefer C to A). S will be sensitive to available means and options for attaining her goals, form intentions on the basis of such options, preferences, beliefs and desires and in normal circumstances reason and act upon those.

Though such minimal rationality is necessary in order to exhibit practical irrationality, it is not sufficient. This has to do with a distinction between two ways of being sensitive to the normativity of reasons for action and belief-formation.^3^ What we need is a requirement that captures the sense in which agents must be sensitive not simply to available means, preference rankings, etc., but more robustly be sensitive to an overall coherence as full-fledged rational agents or persons.

(3) I want to argue that we need a more robust requirement, which both individual and collaborating (and indeed collective or group agents^4^) must, and can, fulfill. I shall label this the *Overall Rational Integrity Requirement*. The central concept at stake, the concept of a *rational unified point of view* (RPV), was introduced by the social ontologist Rovane (1998), who use it to characterize the personal unity of individual and group agents (cf. Korsgaard, 1989; Pettit, 2003a; see more in Szanto, 2014). Though the notion has some structural similarities with the first-person-perspective of subjective experience, importantly, it captures the idea of having a first-person (singular or plural) perspective, but without reference to any subjective phenomenology associated with it (the ‘what-it-is-like,’ if you will, to have that first-person-perspective, or to *be* that person). What then is an RPV? It is a unified set of reasons, in the light of which S assesses her given beliefs, preferences and intentions, and which, in the course of practical deliberation and theoretical reasoning, yields conclusions as to what all-things-considered S *ought* to believe or do. In terms of integrity and autonomy, an agent is an autonomous intentional agent if the agent acknowledges and deliberately endorses the normative practical and theoretical conclusions provided by her own RPV and if necessary modifies her beliefs, preferences or intentions accordingly. This will entail not only a structural or instrumental rationality but also a form of *reflective* rationality. It will entail that agents are aware and deliberatively reflect upon the reasons and motivations they have and act not only *in accordance* with but also *by virtue of* their normative force, or else modify them. Thus, having an RPV is dependent on agents having minimal rationality [hence on (2)], but it furthermore provides the normative force to act in accordance with that structural or instrumental rationality.

When it comes to collaborative contexts and groups, RPV serves both for members and non-members as the basis for the normative and epistemological evaluation of the coherence of shared attitudes or goals. Here, sensitivity to the norms and rational standards of an RPV can be construed in terms of group members’ rational dispositions. These will consist, in particular, in minimizing inconsistencies between the perspectives of the members in view of the pursuit of some shared goal. And it will entail such group-deliberative processes as aiming at majoritarian views, minimizing disagreements or trying to solve them consensually, and, importantly for present purposes, without falling prey to the socio-psychological biases I discuss below (see “Groupthink as a Case of Collaborative Akrasia,” “Further Socio-Psychological Mechanisms Underlying Collaborative SI”). In more complex, especially institutional, forms of groups there will be some normatively binding rational meta-standards for integrating the relevant attitudes. This will include non-contradictory voting procedures and aggregation functions, mechanisms ensuring consistency with other dispositional attitudes, values or sub-goals of the group, predetermined levels of expertise, or even (non-authoritarian) hierarchies in order to rationally evaluate the beliefs in view of group-level goals.

In the following sections, I shall suggest that practical irrationality is typically enhanced in collaborative contexts, and that this is correlated and indeed partly due to disruptions in ER. So first we need an understanding of what ER exactly is and how its mechanisms may be disrupted, particularly in social settings.

## Emotion Regulation and its Social Biases

Psychological work on *individual* ER, or self-regulation, abounds ever since the work of Thompson (1994) and Gross (1998, 2002, 2013)^5^. ER involves the ways in which individuals monitor, modulate or change their emotional elicitation, experience or expression. Less technically, it refers to “influencing which emotions one has, when one has them, and how one experiences and expresses these emotions” (Gross, 1998, p. 271). According to whether ER concerns modulating the primarily situative or dispositional preconditions of emotion-elicitation on the one hand, or the actual emotional episodes or behavioral or expressive effects of those on the other, it is common to distinguish between *antecedent* and *response* focused ER-processes. In particular, ER involves one of the following five processes, or some combination thereof: (i) situation selection; (ii) situation modification; (iii) change of attentional deployment; (iv) cognitive change or reappraisal; and finally (v), on the response-focused side, response modulation. The idea can be brought out by way of the following example. Consider the regular commuter, James, a choleric developmental psychologist, who decides after years of frustration with traffic jams to take the not too crowded train to the office instead. James thus selects a situation in which the likelihood of his anger and frustration is less likely to be elicited (viz. i). One evening on his way back from work, James enjoys his glass of wine and a book in the dining car when a mother with her crying baby chooses to sit right next to him. James feels his anger rising; in order to regulate his emotional upheaval, he might continue to tailor the situation, for example by changing his seat or starting to talk to somebody on the phone with his earphones plugged in (ii). But there are surely more subtle ways of modulating his emotions: For one, James might try to focus his attention on specific perceptual or cognitive features of the situation to alter its emotional impact. He might try to distract himself from the auditory input by looking out the window, or concentrating more on his book (iii). Another possibility of ER is to tell himself or try to make himself think that the book was boring anyway and it’s actually more interesting to observe how mothers interact with young babies outside his university laboratory. Hence, by means of cognitive reappraisal, he might select among alternative meanings attached to the situation (annoying noise vs. interesting ‘field-study’) in order to alter its emotional significance (iv). Finally, if all these mechanisms fail, James would still have a last-ditch move and could modulate the expressive or affective responses and action-tendencies his anger would elicit. He might try to influence his behavioral responses, once initiated, by some props (drinking the whole bottle of wine, or plugging in his earphones and turning the music up to full volume) or expressive behavior (smiling or keeping a neutral expression in order to eventually calm himself down) (v).

However, complex the process, the relevant point for present purposes is that all these dimensions and aspects are robustly shaped by sociocultural factors. Moreover, all these mechanisms and dimensions are often disrupted precisely by these factors. Ample empirical research from developmental, social and cross-cultural psychology supports the thesis that interpersonal or small group settings as well as professional and broader sociocultural contexts robustly shape, modulate or even dictate emotional (self-)regulation (Hochschild, 1983; Kitayama et al., 2004; Parkinson et al., 2005; Mesquita and Albert, 2007; Hofer and Eisenberg, 2008; Mauss et al., 2008; Poder, 2008; Trommsdorff and Rothbaum, 2008; Kappas, 2011; Parkinson and Manstead, 2015). Thus, at the very level of emotion elicitation, affective experiences are often congruent with cultural norms and deeply shaped by interpersonal co-regulation as well as structural and sociocultural affordances (e.g., shame-, or honor-based value-systems, ethnic-pride or caste frameworks) (De Leersnyder et al., 2013). Moreover, emotional reciprocity and reactions to one another also shape groups’ overall “regulatory styles” (Levenson et al., 2014; cf. Maitner et al., 2006). Finally, there is evidence that in large-group contexts with negative emotional exposure (e.g., mass suffering), there are specific negative biases affecting individual ER (e.g., insensitivity, “collapse of compassion”) (Cameron and Payne, 2011).^6^ But even more intriguingly, negative modulations, biases or disruptions in both individual and group-level ER-processes are correlated to irrational tendencies in collaborative deliberations and actions—or so I shall argue.

Thus, just like most other socio-psychological processes emotional co-regulation has not only a bright but also a dark side. Very often it misfires precisely in social, collaborative, intra- and intergroup contexts. And certain collaborative, intra- and inter-group engagements play also a contributing or even constitutive biasing role in entering or maintaining individual as well as group-level practical irrationality. Combining these insights, the guiding hypothesis I begin to explore here is that the negative impact of collaborative contexts on practical rationality is partly due to their specifically disruptive role on ER.

But what exactly is it in ER-processes that social forms of irrationality disrupt? We have seen that ER involves a number of cognitive mechanisms, including situation selection and modification, change of attentional deployment, cognitive change and response modulation. Now, when it comes to assessing *dysfunctional* ER, in an integrative study on the so-called “Difficulties in Emotion Regulation Scale,” lack of emotional clarity and, even more seriously, the lack of emotional awareness has been suggested to be a crucial component (Gratz and Roemer, 2004).^7^ Moreover, it has been reported that the ability to consciously perceive and correctly identify one’s own conative and affective states is key in affect training aimed at better regulating certain negative emotions (e.g., aggressiveness) (Berking and Schwarz, 2014, p. 531ff.). Finally, some have suggested that (self-)deception is a much-used tactic to regulate emotions (Hrubes et al., 2004). Thus, in deceiving others or oneself, one may modify a situation by manipulating the emotions of oneself or others (situation-selection). One may also change one’s cognitive appraisal by means of (self-)deception (re-appraisal): For example, one may convince others or oneself that one’s performance was not so bad after all, or that one’s poor performance was not really one’s fault. Or one may re-direct one’s focus of attention away from particular situations or negative beliefs (attentional deployment): “ideas or beliefs that trigger negative affect may be shifted out of awareness whereas more favorable thoughts or ideas are shifted into awareness” (ibid.: 237–238); or, by means of self-deception, one may try to stop occurring thoughts about negative situations (e.g., situations evoking guilt-feelings), and hence eliminate the guilt-feelings altogether (see also Whisner, 1989).

Building on, further developing or reversing these findings, I want to suggest that certain forms of practical irrationality, and especially DA, inhibit precisely these two crucial features of successful ER, notably *clarity* about the *type*, the evaluative or cognitive *content*, or the more fine-grained *qualitative aspects* of a given emotion. Moreover, they may even hinder the subject to have a *basic awareness* of having a certain *type* of emotion at all.^8^ Below, I will argue that these inhibitions and biases are typically facilitated in collaborative contexts. For now, consider a case illustrating how emotions come into play in *individual* practical irrationality and how practical irrationality eventually disrupts ER.^9^ Mele (2003, pp. 169–170) gives the example of a jealous husband Bob, who fears that his wife Ann is unfaithful. Bob’s fear may be constitutive of his desire that she is innocent and hence plays a role in his self-deceptive behavior to properly assess evidence to the contrary. Not only his fear of Ann’s guilt or desire that she is innocent but also his initial affection may weaken his motivation to assess such evidence (Forgas, 1995). Furthermore, not only might Bob’s emotions increase the probability of the careless assessment of information, but it may even present some information proving Ann’s innocence (e.g., Ann is more affectionate to him lately than ever before) more vividly and saliently than it might upon reflection or to an impartial observer, in fact, be. What are the effects on Bob’s emotion regulation? His self-deceptive behavior will not only result in informational biases regarding the actual facts of the matter but will ultimately lead him to inappropriately assess the evaluative content of his emotional state (‘Well, I have no right to be jealous; everybody is unfaithful nowadays.’), to not fully recognize its phenomenal content and impact (‘After all, I’m not *really* jealous.’), or even to misidentify the very emotion he is experiencing, which he may re-interpret for example in terms of pride (‘My wife is the most attractive woman; everybody always wants to date her.’). In either case, and there may of course be combinations thereof, he will not successfully regulate his jealousy, and will behave, for example, increasingly irritated, nervous or depressive when his wife is not at home.^10^ But how do such irrationally motivated ER-biases spell out in collaboration with others, and how do they eventually enhance practical irrationality to a point where we are left with what I call a ‘spiraling’ of such? This is the question I wish to address in the following section.

## The Collaborative Spiraling of Irrationality

### Social, Mutual, and Collaborative Irrationality

Recall the above discussion of partition of mind accounts of practical irrationality: I have argued that they are insufficient to accommodate the intuition that practical irrationality is not simply about conflicting reasons, intentions or emotions, which are *distributed* across agents or homunculi *within* agents. Here, I want to argue against the related claim that all there is to SI are forms of irresolvable conflicts of interest. In contrast, I shall show that there is an intriguing variety of cases in which irrationality is modulated, facilitated or even triggered by social contexts and collaborative engagements. Before going into any detail, it should be noted that not only such distinctively social but also the overwhelming majority of strictly speaking *individual* forms of practical irrationality are socially co-constituted. More often than not, entering and retaining akratic self-deception or performing akratic actions is facilitated by some social facts, or reactions, deliberative ignorance, or the witting or unwitting assistance by others (Snyder, 1985; Harré, 1988; Ruddick, 1988; Statman, 1997; Landweer, 2001; Tenbrunsel and Messick, 2004; Deweese-Boyd, 2010). In a Sartrean and Marxist spirit, some have even argued that ideology is a form of social ‘illusion’ or self-deception, understood “as the ignorance or the possession of false belief about, [the] social consciousness one has” (Wood, 1988, p. 352).

But leaving aside the issue of the broader social context of practical irrationality that is at play in virtually all cases of irrationality, let me now distinguish three types of the genera of what I shall call *social irrationality* (SI), namely (i) *mutual*, (ii) *communal* and (iii) *collaborative* SI. Far from being a mere exercise in taxonomy, this is crucial in order to get a firm grip on the exact sense in which sociality is or is not involved in modulating the affective and rational life of individuals. In particular, it shall help getting clearer about what it means to properly speaking *collaboratively* engage in practical irrationality.

(i) Consider first *mutual* SI. Suppose two individuals A and B who do *not* engage in any proper collaborative engagement, let alone share any common intentions or goals, but just share a more or less ephemeral situational context. Mutual SI will arise whenever A’s practical irrationality is wittingly or unwittingly assisted or reinforced by B’s appropriate reaction or vice versa. Imagine a patient–doctor interaction in which a terminal-stage cancer diagnosis looms large. In this affectively highly charged situation one or both parties might foreclose or defer an otherwise much more painstaking ER procedure by mutually assisting one another in self-deceptive belief formation about the actual state of affairs. For example, the patient’s self-deceptive report of his actual condition will be facilitated or unraveled during the meeting by the doctor’s (true or false, honest or dishonest) display of an (overly) optimistic attitude, or their *respective* self-deceptive strategies will be reinforced by the behavior of the other (cf. Ruddick, 1988; Trivers, 2002).

(ii) *Communal* SI is similar in structure. The main difference to the mutual case is that the participants are either bound together by a more robust framework of communal, though not necessarily shared interests, habits or policies, or cases in which their respective behavior creates a pattern of ‘quasi-collective’ behavior, for example due to mechanisms of emotional contagion and mutual reinforcement of biasing affects. To illustrate the first scenario, consider a group of professional cyclist who are befriended and all doping. Here the individual cyclists’ akratic or self-deceptive practices are (maybe even unwittingly) assisted by some tacit communal method of concealing certain facts or employing certain habitualized strategies: for example by everybody’s over-optimism (‘Nobody is caught for *this*, come on.’) or euphemistic jargon-talk (‘It’s just a kind of anti-oxidant.’). Notice that there mustn’t be any explicit communal policy or some shared goal that directly motivates SI, as, say for a doping cycling-team (‘We *have* to do this, how else could *we* ever win?’). All there is are some more or less diffuse communal patterns of behavior or discourse. The important point is that, if the individuals were not engaged in the given communal context, they would have a much harder time not just to *rationalize* but simply to be clearly *aware* of *what* exactly they are doing (being akratic cheaters) or to fully realize that they deceive themselves about prospects of being caught. Similar deceptive discourses often facilitate individuals’ irrational behavior in corporate professional settings (Ruddick, 1988; Tenbrunsel and Messick, 2004).

Consider another communal case, a sudden stock-market-meltdown, which is an example discussed by Salmela and Nagatsu (2016) in terms of emotional contagion. Imagine a group of purely egoistically motivated shareholders only concerned with minimizing their *individual* losses. Now, via emotional contagion (Hatfield et al., 2014), behavioral mimicry or similar socio-dynamic processes motivating the individuals’ actions, the individual shareholders’ mass-selling of their own stocks creates an affectively charged situation (a spiral of fear, distrust or ‘collective hysteria’) and results in a quasi-collective behavior, eventually harming *all* shareholders.^11^

(iii) So much then for non-collaborative cases. What I now want to dwell upon are *collaborative* forms of practical irrationalities. Their first distinctive feature is that it involves two or more individuals who are bound together by some collaborative enterprise from the start and collaboratively engage in the very formation, performance or maintenance of an akratic belief or action. There are various scenarios to consider here.

First, consider social dilemmas. They come in many varieties, and I shall only focus on the problem of the commons. But before doing so, as a good way to enter the problem, consider a structurally similar type of irrationality, which although not a genuine case of practical irrationality effectively illustrates how choices, preference rankings or actions may be, individually viewed, fully rational, but turn out to be inconsistent when set into a collective context.^12^ Let there be three subjects with the following three-item preference ranking: S_1_ prefers A to B to C, S_2_ B to C to A and S_3_ C to A to B. Let each individual be fully consistent and sensitive to the transitivity of their own preferences. However, if we aggregate all the rankings by simple majority vote we end up precisely with equal preference rankings for all options, and hence non-transitivity: We then have two collectively aggregated preferences for A to B, two for B to C, and two for C to A. As Hurley puts the point, “collective choices may be irrational despite individual rationality” (Hurley, 1989, p. 138), though this surely doesn’t amount to collaborative practical irrationality of the type we are interested in.

Consider now another social choice problem, individuals’ akratic action concerning natural resource commons. Imagine a fishery in which according to agreed-upon procedures individual fishers must cooperate in order not to over-fish a given sea sector (Ostrom, 1990). Viewed from an individualistic perspective, each fisher has an incentive to maximize one’s own payoff, defect in cooperation (e.g., going out fishing during agreed-upon breaks when others are not), harm the cooperative. If most or all engage in such short-sighted behavior, obviously they will ultimately harm themselves, even though they might profit in the short term. But notice that defecting free-riders, considered separately, will not represent a case of collaborative irrationality. In fact, the rationally dominant choice of free-riders is precisely to defect in cooperation. Viewed from the group level, however, suppose that the co-proprietors fail to agree upon general principles for governing the common together or do not succeed in a robust institutional design for collaborative governance (Dietz et al., 2003). The situation becomes collaboratively irrational if they fail to do so even though they know that in a collaborative framework by failing to do so they risk the resource to dry up and hence are ultimately harming themselves. Even though the members adopt the group’s perspective they fail to reason as “team reasoners” and to act in terms of “team preferences” (Sugden, 2000; Bacharach, 2006)—to wit, preferences that they individually have precisely as co-proprietors of a *common*. This will often, but need not necessarily, happen because of mutual negative influences of individuals’ akratic behavior.^13^

Compare this to another case that involves a properly collaborative *activity*. Consider a similar scenario to that of the doping cyclists above, but with the relevant difference that now we have a genuinely collaborative framework: Let two or more individuals be engaged in some collaborative activity involving a shared goal, collectively accepted beliefs or policies or some similar robustly group-level dimension. Suppose that by agreed-upon policies (all) members of a risk management unit of a bank *jointly* downplay acknowledged high speculative risks, because they individually or collectively aim to maximize profit. In doing so, each member holds the *same type* of akratic action or belief for *similar* or even the *same reasons* and by the *same or similar means.* This may involve a division of labor and hence a differentiation of specific means of irrational behavior. All members being motivationally biased, they jointly deceive themselves. Importantly, as in most collaborative instances of SI, such agency will have more serious negative consequences and result in a negative spiral of lack of self-control and hypocrisy. Akratic actions and beliefs performed in tandem with others may become more easily habitualized and more strongly entrenched as in individual agency, especially when role-models lead by negative examples. But even if this is not the case, the practical implications of one’s own irrational action are usually magnified in collaboration and joint irrational agency (Goleman, 1989; Surbey, 2004). Moreover, individuals’ rational and epistemic control will typically be reduced and individual epistemic responsibility will be weakened or become more diffuse to oneself or others.

### Groupthink As a Case of Collaborative Akrasia

Before I move on, in the next sections, to explain what happens exactly in such collaborative SI, let me finally discuss in some more detail the probably most intriguing case of collaborative (doxastic) akrasia. I will draw upon a real-life small group case study, a clinical group of around 30 would-be non-smokers. It was analyzed by the social psychologist Irving Janis in terms of a paradigmatic instance of what he famously coined “groupthink” (Janis, 1982, pp. 7–8). I will follow Janis’ main thrust but slightly adapt the description of the scenario for reasons of clarity.

Consider then a clinical therapy group of heavy smokers gathering on a regular basis for informal conversation, exchanging views about coping with withdrawal symptoms, motivational advice and clinically supervised medication, in the fashion of an anonymous alcoholic group. At one meeting, a member of the group shyly announces that he succeeded in stopping since the last meeting, an achievement, to wit, that none of the others have attained at that time, or at least have not informed the others about. Now, instead of congratulating him and getting more confident about their own prospects, the other members start slowly, but with increasing expressivity (‘Hey, that’s great for you, but why are you still coming then’?; ‘Not everybody is a hero like you,’ etc.), to treat him as an outsider who deviates from group consensus. In particular, two ferocious members start a heated discussion and voice the claim that smoking is an almost incurable addiction. The debate soon results in a consensus that this is clinically proven. The ‘deviant’ member who has taken issue with the emerging consensus at first realizes quickly that the others have ganged up against him, and eventually declares:

When I joined […], I agreed to follow the two main rules required by the clinic—to make a conscientious effort to stop smoking and to attend every meeting. But I have learned from experience in this group that you can only follow one of the rules, you can’t follow both. And so, I have decided that I will continue to attend every meeting but I have gone back to smoking two packs a day and I will not make any effort to stop smoking again until after the last session (Janis, 1982, p. 8).

Taken at face value, this very sincere, clear-sighted and consistent avowal is followed by the others “beam[ing] at him and applaud[ing] enthusiastically” (ibid.). But the member refrains from quitting the group or leaving the actual session or from reflecting more carefully upon his conflicting desires and emotions (not smoking versus his emotional affiliation to and support by the group, possibly even pride for standing out and succeeding as the only member to stop smoking), in order to modulate his emotions accordingly. The reason is that his deliberative and ER-capacities are overridden by the most salient alternatives and arguments provided by the cohesive social context. Eventually he acts upon these ‘corrupted’ capacities and sticks to the group and smoking.

But there is another side to the story: (Doxastic) akrasia and groupthink do not stop short in exerting their powers, top-down, from group-level to the individual level. As a case of properly collaborative SI, there is a two-way modulation of, or interaction in, performing akratic belief formation and action. As the example clearly illustrates, by the very akratic avowal and behavior of the initially deviant member, the members of the group consider themselves reassured in their own akratic belief that it is impossible to quit smoking all of a sudden. Moreover, there is an affectively motivated irrational tendency of other group members, aggregated on the group-level, to exert pressure on individuals to smoke (even more), especially as the final session approaches,^14^ so as not to lose the affective value attached to the group sessions, such as mutual dependence and affiliation, or in-group solidarity and bonding. But this clearly contradicts the members’ individual goal (to quit smoking) as well as what Tuomela (2007, pp. 32–35; 2013, p. 15) calls the “group ethos” (to help each other to stop smoking, or to this together).

It is important not to misunderstand the notion of group ethos here. To be sure, members do not strictly speaking act *jointly* in pursuing their goal of quitting to smoke. In contrast for instance to the above mentioned case of the risk-management unit or the doping cyclist-team, in this scenario the group does not constitute a group agent proper and hence there is no shared or common goal either. After all, in an obvious sense, it is not the *group* that wants to stop smoking. However, given that we deal here precisely with a smoking therapy group and not just with randomly meeting individual smokers, the framework within which members pursue their individual goals is indeed a collaborative one. This framework represents precisely the group’s ethos. It is constituted by the group’s implicit and explicit rules, shared values and norms, such as the rule to help other members to quit smoking by all means—viz. disregarding purely egoistic motives for (individual) success. As Tuomela puts it, a group’s ethos is something that “functions as kind of underlying presuppositional reason for the participants’ actions” (Tuomela, 2007, p. 34). The notion of group ethos, then, specifies the normative background or rational presupposition for collaborating in light of the members’ integrated rational point of view.

Now I suggest to call the reciprocal, top-down and bottom-up, mutually reinforcing irrational influences outlined here the *collaborative spiraling of irrationality*. Such reciprocal dynamics may happen not only in small groups but just as well in more robust organizational and corporate contexts. There, too, self-deceptive or irrational policies may generate, facilitate or more deeply entrench akratic actions or beliefs in the members. As we have already seen, these policies may include euphemistic or jargon-talk (Tenbrunsel and Messick, 2004) (‘That’s not risky investment; just represent the standard financial challenge to enter the sub-prime real-estate market in emerging countries’), social ramifications of self-deceptive or overly optimistic attitudes. By way of mutual reinforcement, this will result in an overall negative shift in the ‘corporate culture’ of reasoning and acting or to the “corruption” of initially collectively accepted (moral or non-moral) values (Gilbert, 2005; cf. Brief et al., 2001; Darley, 2005). In short, what we see here again is that both parties mutually reinforce akratic tendencies. The group-level irrational tendencies—induced by in-group affiliation, solidarity or social comparison among members—will reinforce individuals’ akrasia, while individuals’ akrasia—induced by the group in the first place—further foster group cohesion, which, in turn, reinforces akratic behavior.

One mechanism that might explain this negative spiraling is due to a structural feature of shared intentions. In his influential account, the social ontologist Michael Bratman has explored this in terms of agents’ “mutual responsiveness” to the intentions and beliefs of one another when they engage in shared agency. As Bratman explains, this responsiveness

Involves [among other features] practical thinking on the part of each that is responsive to the other in ways that track the intended end of the joint activity […] Since the other’s intentions and actions are themselves shaped by her analogous beliefs or expectations, there can be versions of Schelling’s (1980) ‘familiar spiral of reciprocal expectations’ (Bratman, 2014, p. 79).

I want to suggest that it is precisely this spiral that may backfire, as it were, in collaborative SI and cause a negative spiral of reciprocal irrational influences.

In the following (see “Further Socio-Psychological Mechanisms Underlying Collaborative SI” and “Explaining Collaborative Irrationality”), I will discuss further underlying socio-psychological and structural dynamics that help explain what exactly causes the collaborative irrational belief and behavioral tendencies. But for the present case, I suggest that the core mechanism at play is what Janis has described as the phenomenon of groupthink. Janis has shown that groupthink reliably occurs in small- and mid-sized, deeply cohesive groups. As paradigmatic settings for groupthink, he mentions such groups as “infantry platoons, air crews, therapy groups, seminars, and self-study or encounter groups of executives receiving leadership training” (Janis, 1982, p. 7).^15^ In such groups, “members tend to evolve informal norms to preserve friendly intragroup relations and these become part of a hidden agenda at their meetings” (ibid.). Thus, groupthink is characterized by

a mode of thinking that people engage in when they are deeply involved in a cohesive in-group when the members’ strivings for unanimity override their motivation to realistically appraise alternative courses for action. […] Groupthink refers to a deterioration of mental efficiency, reality testing, and moral judgment that results from in-group pressures. (Janis, 1982, p. 9)

What exactly are the defects of a group engaging in groupthink? Janis mentions seven defects in “decision-making tasks” (ibid.: 10): (i) limitation of group discussion to a few alternative courses of action, ignoring alternatives; (ii) lack of surveying the goals and objectives and their implicated values; (iii) failure to re-examine the initially preferred actions regarding non-obvious risks and potential drawbacks; (iv) failure to re-consider courses of action initially deemed unsatisfactory or to consider non-obvious gains or factors that make the chosen alternative appear desirable; (v) lack of any attempt to gain (external) expert opinion about alternatives; (vi) similarly, selective bias to available factual or expert information supporting desirable courses of action and ignorance of external critical views against it; (vii) finally, failure to work out contingency plans to cope with various foreseeable setbacks that might endanger the success of the chosen course of action.

But what are the *structural* and *situational* factors leading groups to such irrational behavior in the first place? Janis suggests a number of “structural faults” of groups fostering groupthink (ibid.: 248–249): they include the above-mentioned group cohesiveness, strong group loyalty and an increasing need for affiliation, especially when facing a crisis situation or being subject to stress; an insulation of cohesive decision-making subgroups from qualified intragroup experts considered as ‘outsiders’ or deviants until the decision is taken; the lack of previously established organizational constraints and norms to adopt methodological checks-and-balances or assess critical information; or an alleged or emerging (false) group norm in favor of a particular action or decision toward which members would feel obliged. Additionally, one of the key, though neither sufficient nor necessary, “situational factors” facilitating groupthink is high stress induced by external factors, such as the “threat of losses to be expected from whatever alternative is chosen and […] low hope of finding a better solution than the one favored by the leader” (ibid.: 250). This explains how groupthink is directly linked to emotion-regulative processes. Arguably, immediate threat of losses or high stress affect not only deliberative but in the first instance affective processes. On the one hand, despair, threats, need for affiliation or stress are prevalent *targets* of ER; on the other hand, they are precisely those type of affective states that tend to heavily interfere and disrupt successful ER.

Now, with a view to the specific focus of this paper, I want to suggest that groupthink, viewed as an underlying, cognitive and affective mechanism facilitating (doxastic) akrasia, arises when members’ intragroup affective bonds and socio-emotional affiliation overrides their motivation or their very ability to rationally assess alternative courses for intentions and actions. What is more, strong groupthink may also inhibit to access one’s own emotional arousal or even some bodily affective states, even those induced by addictive physiological processes. Here then we have a clear case of what I introduced above (see Self-Deception and Doxastic Akrasia) as the *lack of emotional clarity or emotional awareness* that deeply affects ER. At the same time, this cognitive-*cum*-affective corruption leads the members’ capacity to assess alternative ways of ER. Here is how Janis describes this mechanism:

[field experiments indicate] that under certain conditions, increased social contact among group members increases not only the attractiveness of the group but also adherence to norms of self-improvement (for example, giving up smoking). Under other conditions, however, the informal norms that develop may subvert the original purposes for which the group was formed. (Janis, 1982, p. 277)

It should be emphasized that Janis and Hoffman (1970) also discovered the reverse, positive effect of successful non-smoking tendencies due to social affiliation. In a similar small-group setting, they observed members of a group of patients with high-contact partners developing “more unfavorable attitudes toward smoking” and even “fewer withdrawal symptoms of anxiety.” They conclude that “the most plausible mediating factor appears to be the increase in interpersonal attraction produced by daily contact, which makes for increased valuation of the clinic group and internalization of the norms conveyed by the consultant leader” (ibid.: 25). However, in contrast to the cases above, this effect concerns not mutual or group-level but, rather, *interpersonal* influences on ER (i.e., a level of sociality that corresponds to what I have analyzed under the heading of mutual SI). Secondly, it does not negatively affect my main line of argument anyway. Quite the contrary, it adds further ammunition to the claim that even deeply affective symptoms, such as (addiction related) anxiety, are, for better or worse, socially mediated or co-constituted.

### The Corruption of Group-Level Emotion Regulation

But there might be a further concern. One might wonder whether ER-disruptions occur only on the individual level or whether there might be forms of ER that are distinctively *group-level* phenomena. In other words, are there any group-level mechanisms that play the role of ER and eventually has a feedback on the ER-tendencies of individuals, or vice versa. As already indicated, ER processes are not only deeply embedded into socio-cultural contexts; for some tightly coupled pairs of individuals (e.g., infant-caretaker, romantic partners) there are also ER-dynamics at play that can only be viewed as a dyadic ‘loop’ of emotional *co*-regulation (Krueger, 2016; Krueger and Szanto, 2016). But above and beyond that, might *interpersonal* ER result in *group-level* patterns of ER, which may be either disrupted by individual ER-deficiencies, or, conversely, have biasing feedback effects on the latter? I want to briefly argue now that this might indeed be so.

In order to understand these complex socio-emotive interrelations, it is helpful to consider again what has been discussed as *interpersonal* ER (Zaki and Williams, 2013; Parkinson and Manstead, 2015). Interpersonal ER is a process by means of which people are shaping the emotions of others in their immediate social environment. It has been shown that *inter*personal ER positively or negatively impacts *intra*personal ER and vice versa. Making others with whom one interacts feel better heightens one’s own moods (and thus this might be an indirect way of self-regulation), whereas, say, down-regulating one’s own anxiety might lessen concern for oneself by friends (Niven et al., 2012). But interpersonal ER is also an effective manipulator within small-group settings, such as therapeutic support or encounter groups, *viz.* in groups in which there is a high likelihood for groupthink. Thoits (1996), for example, demonstrates how in a psychodrama encounter group the group’s ER-strategies strongly influence the emotions of a targeted individual. Even more strikingly for present purposes, these group-level ER-strategies have significant feedback effects on the group-level display of emotions and the solidarity of the *group as such*. Thoits (1996) describes how the group generates intense negative emotional states in targeted protagonists, ultimately with a view to ‘cathartic personal insight.’ The group uses for example dramatic enactments, teasing, provocations, and non-verbal communication tasks, physical-effort techniques, often enhanced with dramatic music, light effects, etc. In the wake of emotional ‘crashes’ on the part of the individual targets, group members are emotionally affected (“moved collectively” to tears or discomfort) and eventually engage in “group supportive acts.” These involve the collaboration of many participants and include for example collectively displayed acts of bodily comfort to a targeted member (e.g., collaborative lifting, rocking or massaging). Such group-supportive acts of providing ER-aides for an individual would in turn have a feedback on the other members. This happens by means of direct emotional contagion (e.g., seeing someone crying lets the observers breaking out in tears themselves) or vicarious participation (imaginatively taking up the perspective of the targeted protagonist), and leads to group-level display of uplifting affects (e.g., group-wide hugging or energetic dancing). Ultimately, these dynamics produce what Thoits—somewhat hastily, to be sure—calls “shared” or “collective emotions,” increasing group solidarity. Tellingly, Thoits cites a protagonists stating that “‘[It’s not one person’s scene;] it’s *our* scene. This is not just one-to-one work that is being done here; it’s for *all* of us.”’ (Thoits, 1996, pp. 104–105). What we clearly see in this example is that via group-supportive acts the group displays ER-mechanisms that affect individual members’ self-regulation, but also how individuals’ self-regulation has a feedback on the group-level ER process.

The important point is that when ER-processes are disrupted either on the individual or the group level there will also be according ER-feedbacks on both the individual and the group level. And in terms of groupthink, I want to suggest that groupthink might not only lead to individual ER-deficiencies but also individual’s ER-failures might facilitate and reinforce groupthink. Coming back to our initial example of the smoking therapy group, the group-level ER-disruption might deploy as follows: in the face of the akratic disruption of the deviant member and his reinforced irrational behavior—reinforced precisely by the group—the group itself might fail to appropriately regulate its affective dynamics. Due to the affective focus on the deviant member’s threatening the ingroup coherence and the reinforced group-alignment and solidarity induced by groupthink, members may be unable to re-deploy attentional focus away from the ingroup/outgroup dynamics and appropriately assess the new situation, i.e., ‘appropriately’ relative to the initial goal of quitting smoking. They will be incapable of cognitively reappraising the deviancy in terms of actual success, to wit, as a success of the *group* and not just of the member. After all, they might—indeed, according to their rational point of view *ought* to—view the alleged deviancy as exemplifying the group’s emotion regulatory success upon the given individual. And they are unable to view the situation in light of the RPV of the group precisely because this is now corrupted by groupthink, or by increasing ingroup affiliation and outgroup demarcation of the deviant member. Hence, they will fail to act in light of or “promote” their group ethos (Tuomela, 2007, p. 24), according to which the whole *raison d’être* of the group is to collaborate in helping each other to quit smoking, or to do this together.

### Further Socio-Psychological Mechanisms Underlying Collaborative SI

In this section, I want to briefly discuss two further closely related socio-psychological mechanisms underlying SI discussed in literature on choice theory: first, group polarization and choice shift, and second, the pooling of (unshared) information.

Group polarization refers to the widely observed phenomenon that deliberative groups regularly and predictably shift toward more extreme views than pre-deliberation medians, indicated by the members’ pre-deliberation tendencies (Friedkin, 1999; Sunstein, 2002). Consider a group deliberating on whether or not to allow for more social welfare for refugees. Let the discussion members exhibit a representative sample of political views in a given country, ranging from more to less extreme views, for example from the view that ‘refugees should fully pay for the time of their asylum procedure on their own,’ to ‘no additional social welfare,’ or ‘just as much social welfare as for citizens,’ to the most liberal view, ‘more social welfare than for citizens given their precarious state.’ As shown by a number of studies, due to their stronger salience in the group discussion (discussion time, more ferocious voices, etc.), the more extreme views will gain more currency. Eventually, each and every group member will leave the discussion with more extreme views than the mean-range view of the members when entering the discussion (the mean being, say, ‘no additional social welfare’ or ‘just as much social welfare as for citizens’). A similar phenomenon is choice shift. If polled anonymously after a group discussion, individual members of deliberative groups will typically tend to have more extreme positions than the mean initial view (Zuber et al., 1992). The main factors fostering group polarization and choice shift are: (i) a natural tendency toward social comparison among individuals, i.e., the disposition of members to adjust their positions to dominant or salient positions, in order not to stick out too much; (ii) the role of limited, unequally distributed or disproportionate pools of persuasive arguments at the group level, which may push those members who are pre-deliberatively already inclined to the respective views in extreme directions; (iii) inequalities due to interpersonal influences, which emerge during group discussions, for example more or less dominant voices; (iv) finally, and very much as in the case of groupthink, ingroup/outgroup divides and other insulating factors yielding underexposure to views differing from already like-minded ingroup members (Friedkin, 1999; Sunstein, 2002).

Secondly, another cogent phenomenon has often been observed in deliberative groups, namely a certain pooling of (unshared) information (Larson et al., 1994; Stasser et al., 2000). There is a strong tendency, especially in smaller face-to-face discussion groups, to focus on already shared or commonly known and available information, while not taking into account unshared, though maybe highly relevant, information. Again, the pre-deliberation and pre-discussion distribution of decision-relevant information has a strong influence on the content of intragroup and group attitudes, and this significantly affects individual as well as collective reasoning.

As should be obvious, these socio-dynamic tendencies resonate well with the irrational biases and tendencies of groupthink. Moreover, when considering the effect that the salience of views and biased distribution of information has on irrational tendencies, the role of group polarization and the pooling of unshared information in the cognitive and attentional aspects of awareness in ER should also be fairly clear. They will have a key role in corrupting the capacity of group members to change their attentional deployment, and in deficiencies regarding cognitive change or reappraisal. For example, social comparison will push individuals to adjust their views to affects voiced by dominant members (e.g., irrational fears regarding immigration), and a disproportionate pool of persuasive arguments will also make more extreme affects regarding certain positions appear more salient. Thus, these biases will hinder individuals’ emotional clarity, or even lead to a lack of emotional awareness about one’s own or group-level emotional preferences. Just like groupthink, these socio-dynamic processes will significantly modulate or inhibit individuals’ successful ER.

## Explaining Collaborative Irrationality

In the previous two sections, I have mainly focused on explaining which socio-psychological mechanisms are responsible for collaborative SI. What is still missing is a *conceptual* explanation of what exactly happens in such cases. This is the task I wish to pursue in this final section.

To begin with, recall the three requirements that agents capable of practical irrationality must fulfill (see “Emotion Regulation and its Social Biases”). I have argued that it is in particular the Overall Rational Integrity Requirement that is crucial for establishing the possibility of collaborative irrational agency. According to this requirement agents have a unified rational point of view in the light of which they assess their beliefs, preferences or intentions and which, in the course of practical deliberation, yields conclusions as to what, all-things-considered, they ought to believe or do. Now, I want to argue that in genuine cases of collaborative SI—just as in individual cases—the irrationality amounts to a motivated non-conformity to this overall rational integrity requirement. Importantly, the non-conformity is not simply irrational but there is a *motivated* bias—and hence some, if not good, reasons—for the members *qua* members to act or believe accordingly. The motivation deploys its force via the above-mentioned mechanisms and stems from the familiar affective and cognitive biases.

In order to appreciate this point, consider again the subtle but important difference between *mutual* and *collaborative* SI. In the first case the irrational tendencies are also reinforced and facilitated by the interaction with others, but the irrationality of the action or belief remains on the side of the individuals alone. The rationality of the agency, values and beliefs they fail to conform to is determined by the respective individual’s own RPV. And those respective RPV might of course conflict; that is, A’s RPV mustn’t overlap with B’s RPV, and might even be opposed to it. And yet, A might ‘rely’ on B for assisting her not to conform to her own RPV. This is different in genuinely collaborative cases. The irrationality amounts not simply to individuals but to individual *members* not reasoning or acting in light of their *group’s* rational point of view. That is, they fail *as members* to integrate their preferences, intentions or beliefs into the group’s RPV.

Let me explain this in terms of the familiar notion of group identification as it is employed in social identity theories (Hogg and Abrams, 1988). Group identification here refers to a psychological process that involves a cognitive and affective dimension and by dint of which individuals subscribe, acknowledge and indeed phenomenologically experience their group’s perspective, or RPV, *as their own*. Thus, what happens in the cases I am interested in is an irrationally motivated failure of the individuals *qua* group members to integrate their own RPV into the group’s RPV—the group’s with which they keep to group-identify, and that means, whose RPV they keep subscribing to, notwithstanding this failure. Arguably, this is irrational because the members have no good, or no affectively unbiased, reason for *both* identifying with their group’s RPV and their own. But notice that this does not simply mean that the members are sticking to their own preferences, beliefs and intentions, which are in conflict with those dictated by their group’s RPV. Rather, they stick, as group-identifying members, to their own RPV—a point of view, however, of which part and parcel is precisely *the group’s* RPV. Their own RPV would dictate either to quit the group or to stop acknowledging the group’s overall RPV or identifying cognitively and affectively with it. Instead, the individuals keep trying to integrate the group’s RPV into their own, an integration that is doomed to fail, however. But this failure, as I have tried to show, is itself not immediately apparent to the individuals, which explains why they keep trying. The reason for that is that the individual members’ own emotion regulatory processes, and indeed their awareness of their own desires, intentions, and affective states—including their affective attachment to the group—are biased or clouded by the deficiencies of the group’s ER and *vice versa*.

Now, critics may wonder whether such collaborative cases would not simply amount to a certain internal division or partitioning within deliberative groups, such that we end up with conflicting sub-groups or, to put it bluntly, conflicts between what individuals want and what the group wants. Thus, one might think that we are dealing here with a first-order (individual) and a second-order (collaborative) case of irrationality. If so, we would just re-introduce the partitioning picture on a higher level. But this, as I have argued, would not suffice to capture the sense of irrationality at stake in SI. After all, we would then simply be left with competing reasoning or goals and, at best, a compromise of negotiated reasons and intentions. On an alternative construal, DA would either be based on the model of other-deception, where one individual or subgroup deceives another, or amount to a mere problem of practically motivated (epistemic) mis-coordination. The latter possibility might be seen as the result of certain epistemological opacities (maybe due to affective biases), which in turn may result in non-cooperative behavior or the members’ poorly executed coordination regarding a shared goal. But however well they may fit *some* cases, both these explanations seem unable to account for the collaborative nature of irrationality we are looking at.

Relatedly, it might seem that *complete* conformity of the individual’s *overall* rational point of view with that of the group’s is too strong a requirement for group-membership as such. Put differently, does *any* participation in a collaborative endeavor necessarily requires integrating the individual’s *overall* RPV into that of the group on pain of irrationality? This surely seems too hard a bullet to bite in most ordinary cases of collaboration.

Against these potential objections, I want to emphasize again that collaborative irrationality does not amount to a mere problem of an individual and the group or some subgroups having conflicting reasons or interests. Neither do we have one individual or subgroup deceiving another (in which case, again, we would certainly not have any proper *self*-deception or practical irrationality), or one subgroup succumbing to the irrational tendencies of another subgroup. Rather, there is a *temporary disintegration* of collaborative reasoning and deliberation. In other words, what we have here is a motivated, but nonetheless irrational, *disruption of group identification* and eventually *of the overall rational unification of the members*. The result of such will be a temporary disintegration of the rational-*cum*-practical and often affective integration of the individuals, initially bound together by shared reasons, goals and intentions, or a shared affective life. Specifically, what happens is a failure of integrating one’s own rational point of view into that of the group’s rational point of view. But notice again that the failure does not simply amount to a conflict between two incompatible perspectives. For, again, in genuinely collaborative cases, in which the members *identify* cognitively, psychologically or phenomenologically with their group, part and parcel of one’s own RPV is precisely the group’s RPV. Conversely, if collaboration is supposed to be taken seriously—as in therapy group settings for instance—the *group’s* RPV is supposed to be ‘in line’ with their members, and indeed purports to be a more or less practically and affectively coherent integration of each and every member’s RPV. So we don’t simply have two or more RPVs—the group’s and the members’—conflicting with each other but a practically irrational disintegrating force working *among or across* the members and ultimately disrupting the overall unification of the group as a whole.

Viewed from the individuals’ perspective, what goes wrong in the process is that the *relevant* aspect of individuals’ RPV is not sufficiently rationally integrated into the group’s RPV—to wit, the aspect which, via group identification, is in pursuit of a collaborative (and not just an individual) goal. Surely, not in all cases in which individuals cannot integrate their RPV into the group’s do we have individual akrasia or self-deception at play. It might simply be that the individual’s norms of rationality are stronger than the ones dictated by the group or that the group identification of the individual is not strong enough for an irrationality to arise in the first place. In such cases, there is room for “rational deviation” (Gilbert, 2001) from the collective goals and preferences. One will then have rationally valid excuses for not fulfilling the obligation associated with a collective preferences, due for example to conscience reasons. This will be a matter of contextual differences and one will have to look closely into the individual cases. But *if* the individual’s group identification is strong enough, and that means if the group’s RPV is part and parcel of or rationally and *relevantly* integrated into the individual’s, then the conflict will not be a matter of first- and second-order (ir-)rationality conflicting, but itself be a collaborative conflict of rationality. ‘Relevant’ integration here, again, means that the individual must integrate those elements of the group’s RPV into her own that concern *pursuing a collaborative goal* in which the individual aims to partake herself (e.g., ‘*our* goal to stop smoking *together* is *my* goal’). So this doesn’t mean that individual couldn’t *at all*, or *rationally*, deviate from the group’s RPV. She may still try to weigh both the psychological and cognitive evidence for what’s best for *her* to do and what’s best for the *group* (e.g., ‘Is it best for me to stop smoking at risk of tipping the affective in-group balance and dissolving group homogeneity’; ‘Should I not stop smoking and follow the group’s rules,’ etc.). But there are also cases—and these are the ones I have been discussing—where one may not have rationally valid excuses or reason (for oneself) *not* to fulfill the obligations associated with a collective goal. Moreover, because the individual is torn not only by her own akratic tendencies but also, and more importantly, given her initial group identification, biased by in-group affiliation tendencies or similar dis-regulatory mechanisms, she might simply not be any more in a position to rationally (what is all things considered best to do) and also psychologically (what is best for me given my group identification and my own goals) adequately assess the available evidences.

Similarly, on the group level, the disintegration is not simply a partitioning of the group into conflicting subgroups, but rather a dissolution of the normative force of an initially *joint* commitment to collective reasons, values and practical conclusions, which are provided by its rational point of view. That is, not only do all of the above requirements (1)–(3) still hold, such that the irrationality can arise in the first place. What is more, the members acknowledge and also continue to lay claim to their group’s rational point of view, such that its normative force still exerts its influence.

Another way to put this, is to point to a certain disintegration or drifting apart of individuals’ ‘personal’ intentions, on the one hand, and the group’s ‘joint’ intention, on the other. In terms of Gilbert’s (1989, 2009), prominent account of collective intentionality this may happen due to the fact that—being motivationally biased—*none* of the participants of a certain collaborative endeavor has a *personal* commitment to a shared belief or intention, even though they continue to be *jointly* committed to it. To appreciate this point, consider Gilbert’s notion of ‘joint commitment.’ Gilbert uses this notion as a technical concept to highlight a difference between ‘joint’ and ‘personal’ commitments to collective beliefs or actions. In her view, when parties jointly commit themselves to a shared belief or intention, they must see to it “as far as possible to emulate, by virtue of the actions of each, a single body that intends to do the thing in question” (Gilbert, 2009, p. 180). By doing so, they are jointly committed to the intentional action. The central idea is that in sharp contrast to *personal* commitments none of the parties can suspend the normative force of the commitment thus created *individually* or separately but, rather, precisely only through *joint* deliberation. But as I have tried to argue, what happens in collaborative SI is precisely the opposite: The parties, due to collaboratively induced ER-deficiencies and the ensuing affective and cognitive biases, do not properly realize that they in fact are still *jointly* committed to the collaboration, while they only act upon their (akratic) *personal* intentions.

## Conclusion and Future Directions

I have argued that given three requirements agents are capable of mutual, communal, and in particular collaborative forms of AA and DA. I have provided a conceptual model to analyze these in terms of a failure to integrate individual members’ rational point of views into the overall rational point of view of the group with which the members keeps to group-identify. Indeed, I hope to have shown that such social forms of irrationality are not only real but are also rather common and prevalent phenomena. Moreover, I have emphasized that in collaborative cases there is a two-way modulation, a bottom-up, leading from individuals’ irrational tendencies to that of the group, and a top-down, leading from groups’ irrational policies to individuals’ irrational action and belief-formation. I have explored this reciprocal, reinforcing dynamics in terms of what I call ‘collaborative spiraling of practical irrationality.’ Furthermore, I have argued that in some instances collaborative irrationality is due to a salient deficiency in ER, namely to the socially motivated misidentification of one’s own affects. I have suggested that this biases one’s own affect control and eventually one’s group’s ER. Consequently, I have claimed that various social engagements often play a contributing or even constitutive role in entering or maintaining practical irrationality, and this is, in turn, partly due precisely to their disruptive role on ER.

Now, even if the argument goes through, some may wonder whether there would lurk a circularity at its very dialectical core, and especially when considering the latter claims: Thus, it might seem that there is a circularity between the claim that some social forms of irrationality have a disruptive role on ER, on the one hand, and the claim that the resulting biases in individual and group-level ER are reinforcing collaborative irrationality, on the other. Put differently, one may wonder about the direction of the causal influence regarding the emergence and indeed intensification of irrational biases: is it failures in emotional (co-)regulation leading to or increasing collaborative akrasia or vice versa? Or does the influence go both ways? However, I believe that the ‘circularity,’ though in fact real, represents a virtuous and not a vicious circle, which rather than threatens the argument, lends credit to it. For, indeed, there is a certain feedback between disruptions of collaborative and individual rationality, on the one hand, and disruptions of individual and group-level ER, on the other, and this mirrors the spiraling of collaborative irrationality that I have elaborated upon. Typically, in real-life scenarios, once this spiraling is set in motion, it proves almost impossible to stop the feedback-loop between disruptions of what one or one’s group can emotionally access and regulate, on the one hand, and disruptions of what one or one’s group is rationally able to do or to think, on the other.

Let me close with a remark on a lacuna and the potential direction for future research. In this article, I have concentrated on mutual, communal, and especially collaborative forms of practical irrationality. However, I contend that given our three requirements there is also room for genuinely *collective* and *organizational* forms of SI. The subjects of akrasia and self-deception would then not be collaborating *individuals* but fully fledged group or corporate agents (Pettit, 2003a; cf. Sugden, 2012). But if one admits such cases, one might also wonder how collective or shared emotions might play a role here and, furthermore, whether there might be not just interpersonal and group-level but genuinely collective forms of ER-biases. Though this is still contentious and depend on a number of further assumptions about the possibility of collective agency and emotions, there is already a large body of literature in philosophy that may path the way to move ahead in this direction (e.g., Schmid, 2009; List and Pettit, 2011; von Scheve and Salmela, 2014; Szanto, 2015, 2016, 2017; Tollefsen, 2015; León et al., under review). Above and beyond the need to properly analyze these cases in and for themselves, I believe that in order to get clear about the exact sense in which sociality is modulating the affective and rational life of individuals, future research should analyze this whole variety of cases. Only then shall we adequately understand the sense in which we not only help regulating our emotions in tandem with others or together but also the sense in which ER and rational behavior systematically fails precisely given the presence of others.

## Author Contributions

The author confirms being the sole contributor of this work and approved it for publication.

## Conflict of Interest Statement

The author declares that the research was conducted in the absence of any commercial or financial relationships that could be construed as a potential conflict of interest.

## Footnotes

1 Throughout the paper, my argument will build on small-group or organizational settings. I will not discuss the broader sociocultural scaffoldings facilitating affective modulations and emotion-regulative disruptions. For such an account, see Collins’ (2004) congenial sociological analysis of the broader socio-normative context of so-called “tobacco rituals and anti-rituals” and the way in which they are scaffolded by material and bodily culture (e.g., smoking lounges, advertisement culture, gestures, etc.), and co-constitute the regulation of smoking pleasure and displeasure, as well as the very experience of tobacco enjoyment, and eventually the very psycho-physiological effects of addiction.

2 I cannot argue here against analyses of akrasia in terms of a change of mind or change of volition, see McIntyre (2006). However, I come back to the partitioning of mind accounts below.

3 For similar but ultimately different distinctions between the ways in which an agent is sensitive to (the normativity of) practical rationality, such as “guidance condition” and “motivation condition,” see Wallace (1999a, pp. 217–219); see also Jones’ (2003) distinction between “reason-responders” and “reason-trackers” and, again, Döring (2010).

4 For the purposes of this article, however, I am not committed to the claim that groups as such, or group agents are constituted by having an own RPV (see Szanto, 2014) and accordingly may be proper agents or subjects of practical irrationality (Pettit, 2003b); see also the Section “Explaining Collaborative Irrationality.”

5 See on emotion regulation in different developmental, social and non-social settings (Thompson, 1994; Campos et al., 2011; Varga and Krueger, 2013; Levenson et al., 2014; Krueger, 2016; Taipale, 2016).

6 Interestingly, however, there is hardly any work on how co-regulation may ‘scale up’ so as to include not only dyads and face-to-face groups, or one’s sociocultural context, but also ER-processes of large communities or nations, e.g., in the wake of large-scale traumatic events such as the Katrina or Fukushima disasters, the present European ‘refugee crisis,’ or 9/11 (cf. Levenson et al., 2014, p. 279).

7 To be sure, some have recently argued that the picture looks somewhat different when it comes to addiction, where not all types of self-knowledge about one’s own addiction (e.g., first-, third-personal, critical, impersonal self-knowledge) are equally apt to improve self-control; see Levy (2014, 2016), Holton (2016), and Morgan and O’Brien (2016); for empirically well-informed research on addiction and self-deception, which is congenial to my argument, see, however, Pickard (2016). For further useful philosophical accounts of addiction and irrationality, however, different they may be (see Wallace, 1999b; Schlimme, 2010; Uusitalo et al., 2013). For a review of literature on emotion regulation in drug abuse, see Kober (2014).

8 I certainly cannot enter here into the complex discussion of whether one can have emotions that one is unaware of having or the issue of non-felt emotions. Suffice it to say that I agree with Roberts that one can indeed have both emotions that one does not feel and emotions that one is unaware of having (Roberts, 2003, pp. 60–69, 318–323). In any case, the point I’m trying to make here is orthogonal to that issue. I want to put forth only the more modest claim that one can be *unclear* or *confused* about certain emotional aspects or unaware of the *content* or *type* of a *given* emotion. Hence, my concern here is only with types of emotional error or misrepresentation induced by practical irrationality (see more on this below, and again Roberts, 2003, esp. Chap. 4). For useful discussions of the veridicality, correctness and justification of emotions, see Deonna and Teroni (2012, chapters 1, 4, and 8).

9 I will not argue for the claim here that in some or maybe all cases of practical irrationality emotions play a direct or indirect biasing role; see Mele (2003). Let me just mention that there is a vast body of empirical literature demonstrating that emotions often prime cognitive faculties in gathering and assessing evidence in a biased way (Nisbett and Ross, 1980; Derryberry, 1988; Forgas, 1990; Kunda, 1990; Dalgleish, 1997; Trope et al., 1997; Tiedens and Linton, 2001; Schwarz and Clore, 2003).

10 Here, another caveat is in order: When arguing for the role that emotions play in practical irrationality, I shall not engage in discussions about whether emotions as such can be or are in general rational (de Sousa, 1987; cf. De Sousa, 1978), or whether, conversely, emotions or emotional (in contrast to rational) choice are, typically or necessarily, at the base of motivational biases in practical irrationality (Elster, 2010). Moreover, I will not take any stance on the intriguing question of whether and how emotions are indispensable for rational agency, namely, whether emotions are, positively viewed, an own source of practical knowledge (Döring, 2010) or whether they are instead non-rational sources of action-eliciting desires (Elster, 2010). Finally, I wish to remain neutral on the relation between emotions and the role of action-eliciting, action-causing or the motivational power of desires as standardly conceived in folk-psychological accounts of action (see Deonna and Teroni, 2012, chapter 3).

11 There is a slightly different but cogent case discussed by David Lewis in *Convention* (Lewis, 1969, p. 87) in which a self-deceptive agreement on conforming to a convention ultimately destroys the normative force of the convention and hence hinders coordination.

12 Cf. again a similar but distinct case of a so-called discursive dilemma as discussed by List and Pettit (2011; see also Pettit, 2003b). Notice, however, that discursive dilemmas are not irrational in any of the discussed senses here; cf., however, Sugden (2012).

13 Cf. also Gilbert (2001), who convincingly argues against Sugden (2000) for the stronger claim that “collective preferences” give sufficient normative reasons for the members of a group to act in the light of those preferences or even “obligate” them do so. That doesn’t mean that members could not “rationally deviate” from group preferences. But if their reasons are not based on *rational* deliberation of what is best to do in light of those collective preferences to which they have initially committed themselves, then the group is “entitled” to “rebuke” the member. And even if based on rational deliberation, still the members are obligated to an explanation and indeed an apology of why they are not acting in the light of the collective preferences. See more on the implications of Gilbert’s theory for my argument below, see the Section “Explaining Collaborative Irrationality.”

14 This has been empirically well documented; for further social influences on smoking cessation see Forsyth (2001, p. 635), Quintana (1993), and Collins et al. (1990).

15 For more examples, see the review-essay Esser (1998).
